# Children’s Experience of Symptoms: Narratives through Words and Images †

**DOI:** 10.3390/children5040053

**Published:** 2018-04-19

**Authors:** Barbara M. Sourkes

**Affiliations:** 1 Division of Pediatric Critical Care Medicine, Stanford University School of Medicine, Palo Alto, CA 94304-5876, USA; bsourkes@stanford.edu; 2 Pediatric Palliative Care Program, Lucile Packard Children’s Hospital Stanford, Palo Alto, CA 94304-5731, USA

**Keywords:** pediatric palliative care, life-threatening illness, complex chronic illness, symptoms, experience, psychological, trauma

## Abstract

Children who live with a complex chronic or life-threatening illness face extraordinary challenges. Whether they are receiving disease-oriented treatment (aimed at potential cure or prolongation of life) or palliative treatment—or both concurrently—our challenge is to enhance their comfort and minimize their distress. Symptom management is thus a critical component of pediatric palliative care. Symptoms may be either physical or psychological in nature (or a confluence of both) and their effective management has a direct impact on the child’s quality of life. This article provides an integrative overview of children’s experience of selected physical and psychological symptoms, as expressed through their words and images. Understanding their perspectives is an essential component in the design and provision of optimal symptom management. Included, as well, are examples from siblings—a reminder of the profound impact of illness on these children who also “live” the experience, albeit in a different way. The symptoms that are described are pain, nausea and vomiting, fatigue, weakness, seizures, hair loss, depression, and anxiety. Although psychological symptoms are often inextricable from the physical, they may also present independently as part of the overall illness experience.

## 1. Introduction

Children who live with a complex chronic or life-threatening illness face extraordinary physical and psychological challenges. Many contend with hardship and terror as the illness wends its course into an uncertain future. Pediatric palliative care strives to optimize the quality of life of children living with serious illness, as well as to support the family. Whether children are receiving disease-oriented treatment (aimed at potential cure or prolongation of life) or palliative treatment—or both concurrently—our challenge is to enhance their comfort and minimize their distress.

The concept of psychic trauma lends itself to understanding children’s experience of illness. Terr, a child psychiatrist, offers the following definition in her book *Too Scared to Cry*: “‘Psychic trauma’ occurs when a sudden, unexpected, overwhelmingly intense emotional blow or a series of blows assaults the person from outside. Traumatic events are external, but they quickly become incorporated into the mind. A person probably will not become fully traumatized unless he or she feels utterly helpless during the event or events.” [1] (p. 8). This description certainly relates to the overwhelming sense of loss of control experienced by seriously-ill children: the shock of diagnosis, the indelible imprint of the sustained assault on the body and psyche, and the uncertainty of the outcome. Whereas “external” malevolence of intent characterizes many forms of trauma (e.g., abuse), the culprit in illness resides within the body itself and often in the inexplicable randomness of fate [2] (p. 4).

While many physical symptoms are a predictable manifestation of an underlying disease or disorder, the intensity and frequency of their occurrence can be highly variable. Psychological symptoms are often not as predictable and, as a result, may take longer to identify and address. Illness unfolds within the broader context of the “whole” child and family: thus social, cultural, and religious factors may all have an impact on how a child experiences and interprets a given symptom [3].

This article provides an integrative overview of children’s experience of a range of physical and psychological symptoms, as expressed through their words and images. Understanding their perspectives is an essential component in the design and provision of optimal symptom management. Included, as well, are examples from siblings—a reminder of the profound impact of illness on these children who also “live” the experience, albeit in a different way. Although only the patient experiences the physical aspects of illness, the reverberations on the siblings may become a source of great distress, especially because they often go unacknowledged.

Many children in pediatric palliative care have diagnoses with cognitive, as well as physical, manifestations. The spectrum includes children with mild/moderate limitations to those with severe global developmental deficits and minimal awareness of the world around them. In addition are children who are cognitively normal, but may not be able to communicate effectively during certain phases of the illness. Thus, the children who “speak” in this article may be seen as the expressive voice for many others who suffer similar symptoms but are unable to report their experience.

The selected symptoms to be described are pain, nausea and vomiting, fatigue, weakness, seizures, hair loss, depression, and anxiety. Although psychological symptoms are often inextricable from the physical, they may also present independently as part of the overall illness experience.

## 2. Pain

The term “pain” [4,5,6,7] has many meanings, ranging from the physical to the psychological and, most often, a confluence of the two. In this section are examples of stark depictions of physical pain, bearing pain for the sake of living, and pain associated with death.

### 2.1. Depictions of Physical Pain

Psychologist: If you could choose one word to describe the time since your diagnosis, what would it be?

Child (without hesitation): PAIN. Once I felt as if an I.V. was exploding in my arm!

[8] (p. 23)

**Figure 1:**
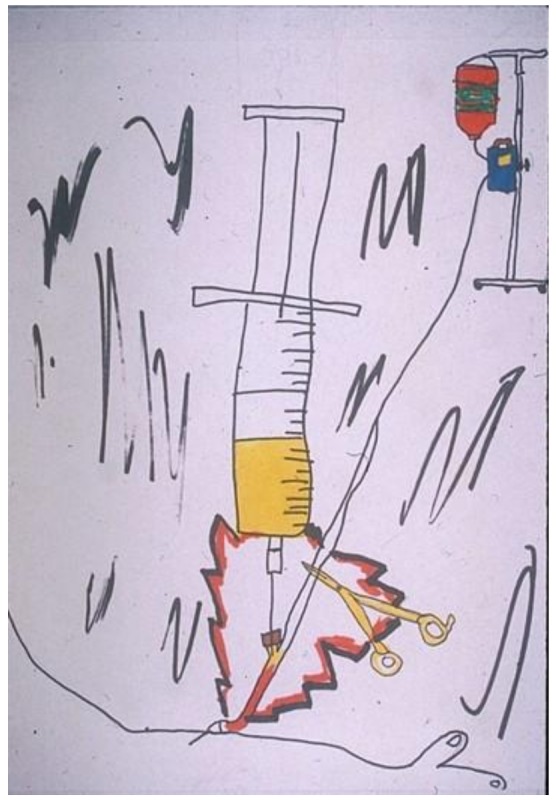
Intravenous (IV) exploding in my arm.

The child then described the excruciating pain he had felt when someone tripped over his IV pole that then came crashing down (Figure 1). The boldly colored, nightmarish image with foreboding slashes of black conveys the extreme pain and the associated anxiety and vulnerability. At the bottom of the picture—totally overwhelmed by the chaos—is his arm, the site of the pain.

**Figure 2:**
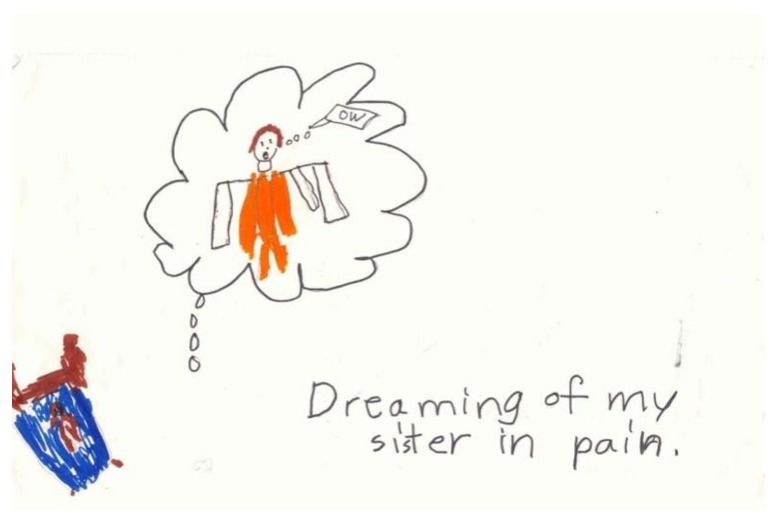
Dreaming of my sister in pain.

In response to the question: “What is the scariest feeling, thought or experience you have had since you sister became ill?” a healthy sibling drew her response: “Dreaming of my sister in pain….” (Figure 2) [8] (p. 23). She depicted herself as a diminutive brown figure in a small bed, overwhelmed by the dream image of her sister in bright orange—screaming “OW.” Her image testifies to the powerful impact on siblings of witnessing the patient’s suffering. Even as they are spared the physical pain of illness, they are unshielded from a sense of fear and overwhelming helplessness. Therein lies the source of the siblings’ trauma, in counterpoint to the patients’.

### 2.2. Bearing Pain for the Sake of Living

Children are well aware that, despite everyone’s best efforts, certain life-prolonging or curative treatments may cause them distress or pain. They can perceive the cost/benefit exchange: what they are willing to withstand on an ongoing basis in order to achieve the goal of living.

**Figure 3:**
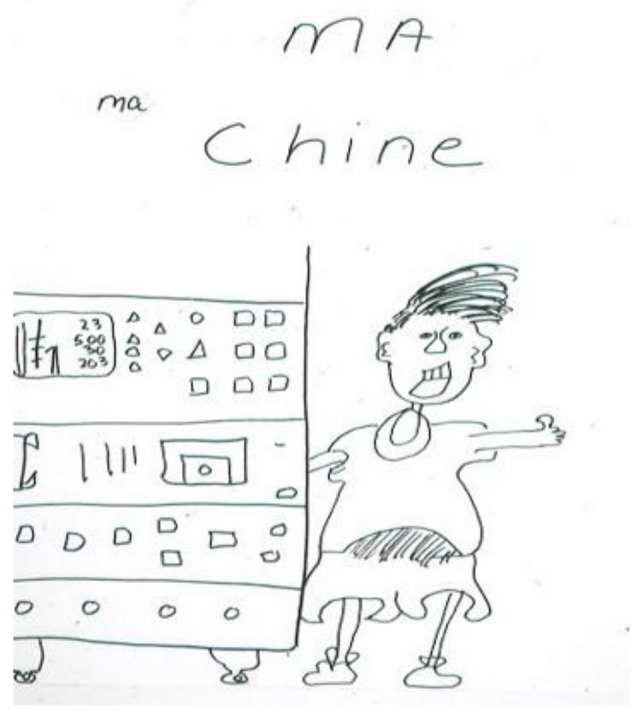
MA machine.

A teenager with end-stage renal disease depicted his absolute dependence on hemodialysis to live. He complained about many levels of discomfort: from the disruption to his life caused by the dialysis schedule to increasing pain associated with the treatment itself. He entitled his drawing (in French) “MA machine” (“MY machine”). (Figure 3) [8] (p. 23), with the emphasis on “MA/MY”. The possessiveness is an indication of its critical importance to him in sustaining his life. One hand is literally plugged into the machine, while the other is in a “thumbs up” gesture. His facial expression is ambiguous—triumph, horror, or a combination of the two.

### 2.3. Pain Associated with Death

**Figure 4:**
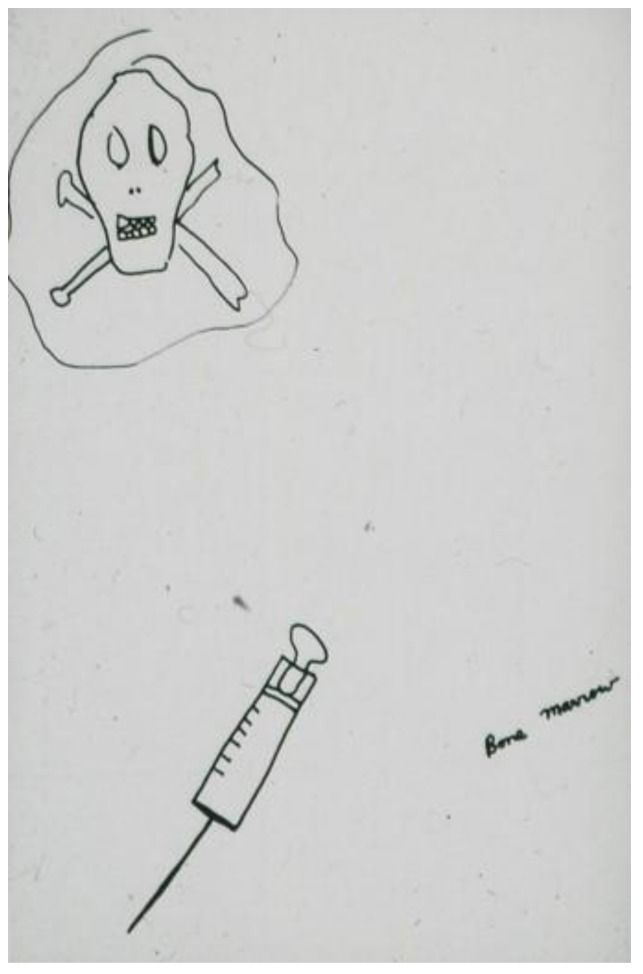
Bone marrow aspiration needle.

**Figure 5:**
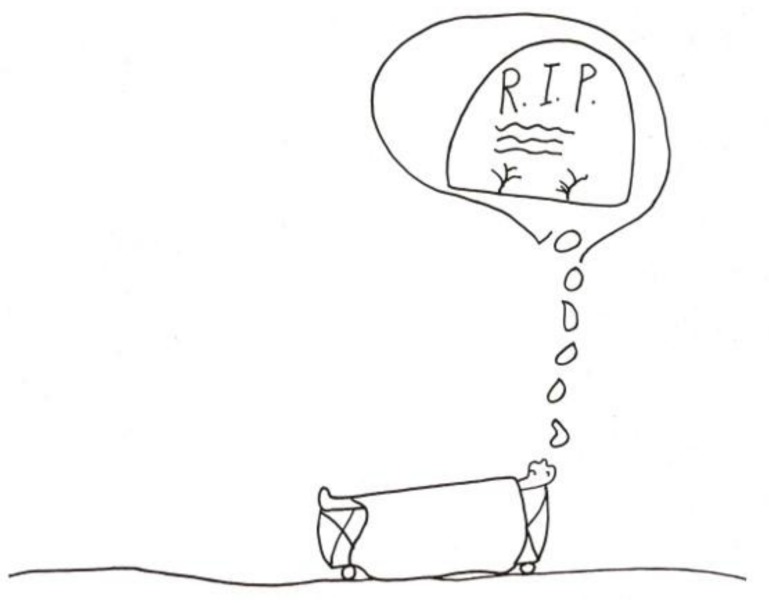
Tombstone.

For some children, the experience of pain is directly associated with the fear of death. This occurs even in children whose prognosis is good, and although death is a threat, it is by no means a certain outcome. The child who drew the image of the skull and crossbones leering above a bone marrow aspiration needle (Figure 4) [2] (pp. 114–115) had an excellent prognosis. In an image that *explicitly* links pain with the thought of death (Figure 5) [2] (pp. 114–115) a child stated: “When I’m just lying in bed in the hospital and in pain, this (pointing to his drawing) is what I think about.” (R.I.P. stands for “Rest in Peace”).

## 3. Nausea and Vomiting

Nausea and vomiting [9,10] can be highly stressful for children on many levels. Very young children may be frightened by the unpredictability of vomiting and retching. Older children respond to the pervasive (and intrusive) nature of nausea: “I can’t even think straight when I feel like throwing up. It blocks out everything.” Or, as a sibling stated matter-of-factly: “I hate when he pukes all the time. It sounds bad and it stinks.” The fact that nausea is antithetical to having an appetite for food can be distressing for both children and parents. On a “medical” level, there is the worry about the child not getting adequate nutrition. On an emotional level, nausea precludes food as a source of pleasure and comfort for a child, as well as taking away an important avenue for parents to provide that comfort.

**Figure 6:**
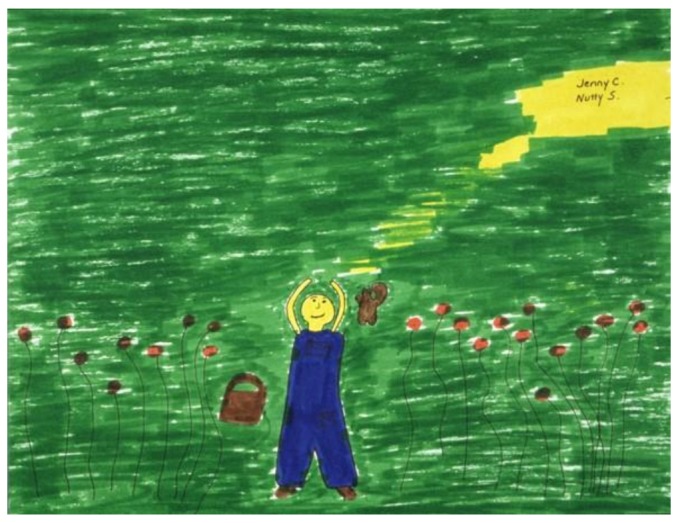
Relaxation scene.

In preparation for learning relaxation/hypnosis techniques as an antidote to nausea, a child composed the following image involving her favorite stuffed animal, Nutty the Squirrel. Like many children, she yearned for the nausea to end so that she could eat her favorite foods (Figure 6): “This is me lying in a field of flowers on a warm, sunny day with Nutty the Squirrel. I am barefoot, wearing overalls. We would have a picnic basket filled with both our favorite foods: nut soup, chicken rice soup, sandwiches, lasagna, cookies, candy, ice cream, apples and milk.” [2] (p. 63).

## 4. Fatigue

Fatigue [11]—a child’s complaint of “always feeling tired”—is now recognized as an independent symptom in and of itself, rather than simply being taken for granted as a “given” of the effects of illness or activity. For adolescents who want to be out in the world with their peers, fatigue is a huge impediment to their quality of life. In the words of a teenager: “When I am feeling sick, I am sick—that’s it. But when I am feeling okay, I hate being too tired to do anything. It makes me feel like an old person”.

Fatigue can also be an intrinsic part of the dying process, and in that context, it may actually bring a measure of peace.

**Figure 7:**
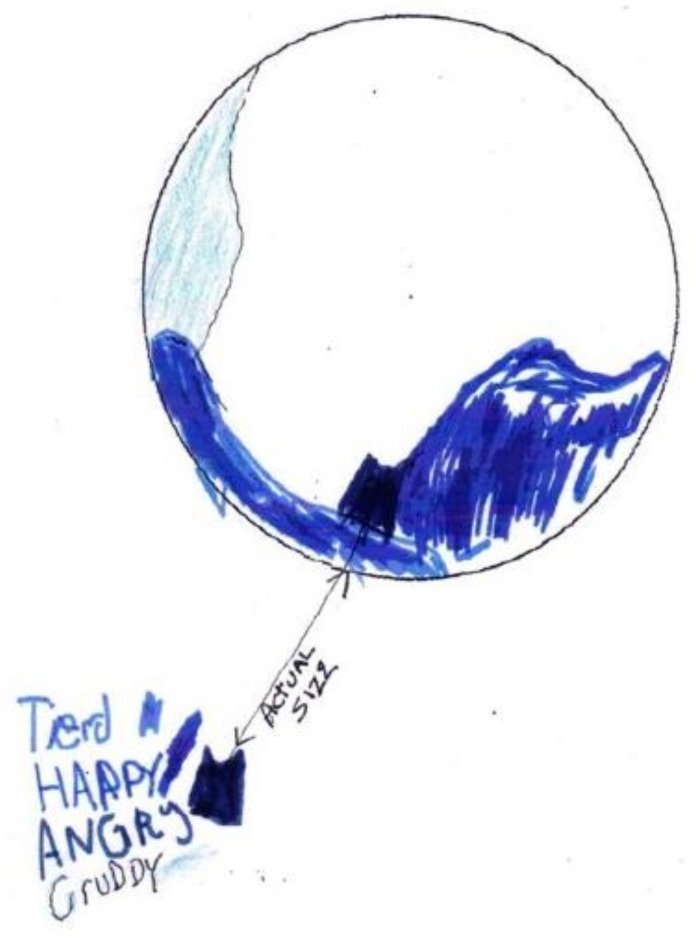
Tired.

A child did her last drawing, a color-feeing mandala, four days before her death (Figure 7) [12] (p. 31). She was calm and deliberate, although very weak, as she described “How I am feeling today: tired (medium blue), happy (medium—deep blue “wave” to right of tired), angry (dark green inset), cruddy (pale blue).” Her one comment on the deepening shades of blue in her mandala was: “I wanted to put in more ”happy” but I am too tired to draw anymore… I am happy with my family.” She spoke quietly and smiled. Her image and words provided the opening for a conversation with her parents, who told her that they recognized her tiredness, and that it was all right for her to “let go”.

## 5. Weakness

While generalized weakness may be a symptom of many diseases, it is a very specific symptom (and omen) in degenerative disorders.

**Figure 8:**
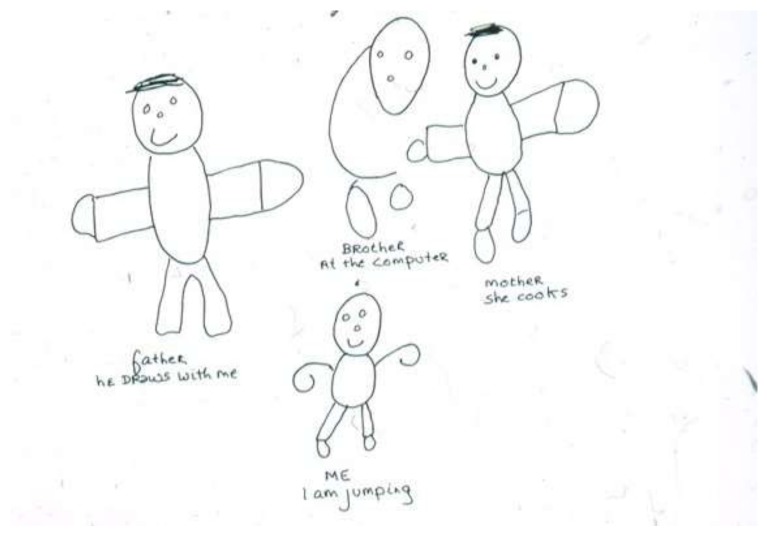
I am jumping.

“A child with muscular dystrophy was tripping and falling constantly, but adamantly refused to use a wheelchair, protesting that he did not need it. His older brother with the same disease was already severely compromised. In a family drawing, (Figure 8) the child portrayed himself jumping and smiling; he drew his brother as an incomplete almost ghost-like figure at the computer. The extremities of all four family members are distorted or missing. This child’s awareness—and attempted denial—of his own progressive deterioration as well as his brother’s status (and thus his own in the future) are embedded in the drawing” [8] (p. 25).

**Figure 9:**
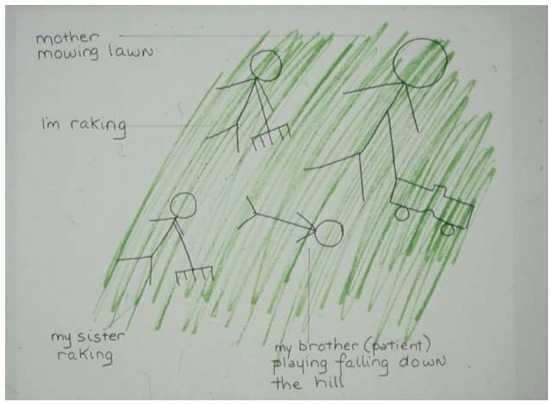
My brother playing falling down the hill.

In a thematically-related drawing (Figure 9), a child from a different family drew her mother, her sister, and herself working in their hillside yard; she described her brother as “*playing* [author’s italics] falling down the hill.” Although she had been told that his weakness was actually a symptom of disease, she was still hopeful that it was not of any serious import.

## 6. Seizures

**Figure 10:**
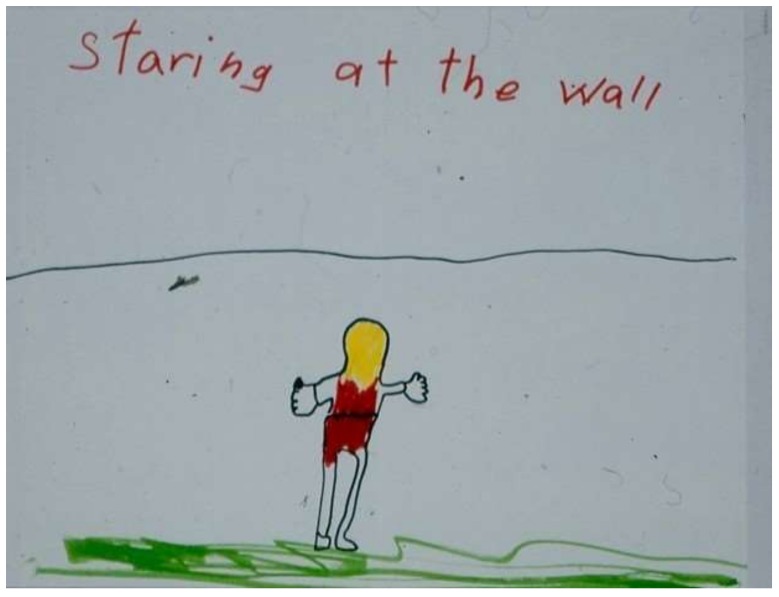
Staring at the wall.

Seizures [13,14] are a symptom that—over and above their physical properties—carry a great deal of emotional, social, and cultural impact. For children with severe neurological deficits and minimal awareness, the primary focus is on medical management of their seizures. However, for children who are more functional, the unpredictability of seizures can undermine their confidence in venturing out in the world. They are aware that the dramatic presentation of some types of seizures is frightening and mysterious to others, and they fear social stigmatization. As a sibling of a child with absence seizures (petit mal) reflected (Figure 10): “I hate when my sister suddenly stares in the middle of us playing or talking. It makes her weird”.

## 7. Hair Loss

**Figure 11:**
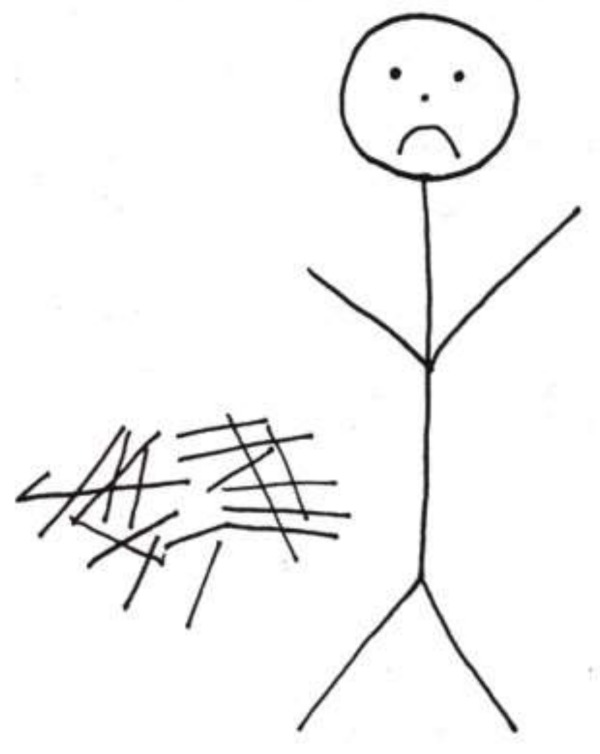
Losing my hair.

Acceptance of the temporary hair loss secondary to chemotherapy is initially difficult for most children, regardless of age. Reactions of fear and sadness are common. Even young children recognize its significance, from the cosmetic surface through to the evidence of the gravity of the illness. A child stated that losing her hair been the hardest part of the illness for her (Figure 11) [2] (p. 45). She portrays herself in her drawing as stripped and vulnerable.

Over time, matter-of-fact comments about the baldness—even humor—surface.

**Figure 12:**
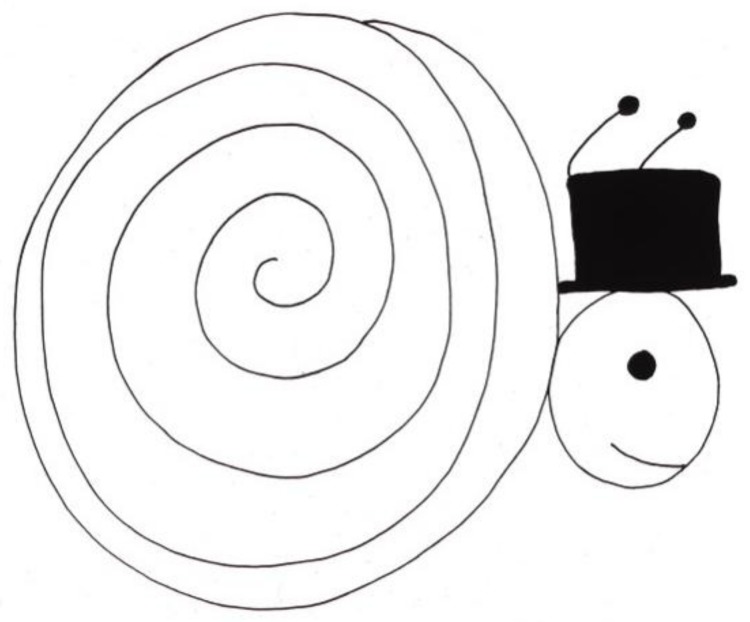
Mr. Snail.

A child who had been bald most of her life because of continuous treatment stated: “I don’t have hair because I take very strong medicine. Sometimes people laugh at me. It’s not very fair. It makes me mad.” She proceeded to draw a picture with the following explanation: “This is Mr. Snail, the groom. He is going to his wedding. He had to brush his hair, so he’s late.” (Figure 12) [2] (p. 46). Her identification with the snail was clear, since her own head was smooth and shiny and often covered with an interesting hat.

## 8. Depression and Anxiety

Depression and anxiety [15,16] were once considered as an inevitable part of children’s reaction to serious illness and were accorded little attention. Fortunately, these psychological symptoms are now recognized as symptoms in and of themselves that are as important to address as physical distress. Psychotherapy and psychotropic medication may become part of an integrated treatment plan.

“Although many psychological problems may be categorized as adjustment reactions, more severe psychopathology can emerge. This is especially true in children with preexistent vulnerabilities, or when there is a prior psychiatric history in a family member. While it is important not to overemphasize psychopathology, there is also a risk in minimizing or not recognizing it. Any psychological response, however benign initially, can develop into a more complex symptom under the sustained stress of illness. Thus, the severity of symptoms, particularly in terms of intensity and duration, must be continually assessed relative to the child’s current reality” [2] (p. 9). Furthermore, psychological symptoms often present in ways that, at least initially, appear indistinguishable from physical distress (e.g., fatigue may in fact be indicative of depression). It often takes highly skilled medical and psychological evaluation to differentiate the source of the distress.

Children’s drawings can be clues to identifying psychological symptoms.

**Figure 13:**
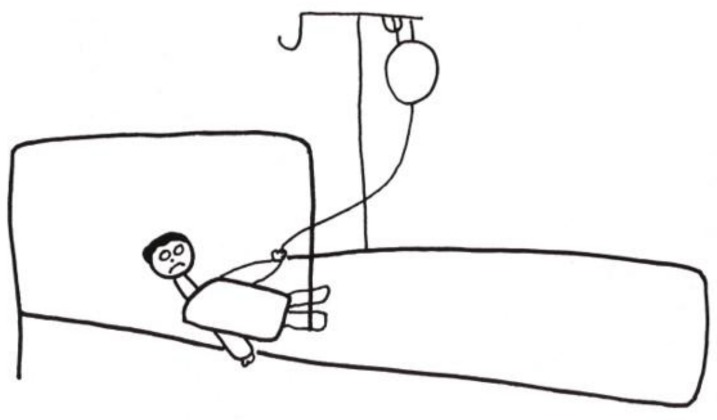
Sad boy in the hospital.

**Figure 14:**
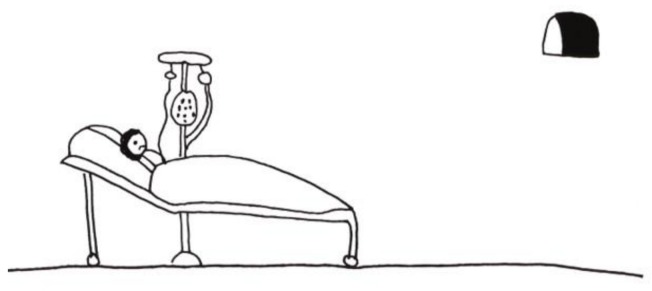
Alone in the hospital.

**Figure 15:**
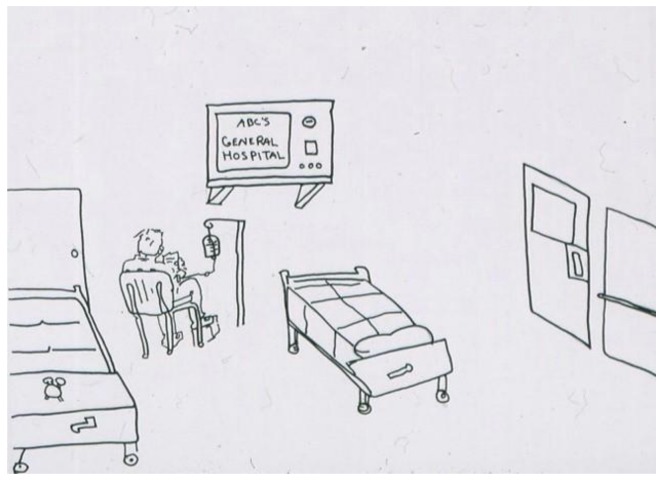
Being in the hospital.

The following two images drawn by hospitalized children (Figure 13 [2] (p. 64) and Figure 14 [2] (p. 65) are striking for their emptiness and isolation. In Figure 14, the stark isolation is ‘relieved” only by an ominous-looking television. Contrast these two images with Figure 15. Although this artist was older (an adolescent), it is not only age that accounts for the difference in what his drawing communicates. Despite being alone in the hospital room, there is a sense of involvement in life. The door has a window, there are two beds suggesting the possibility of a roommate, and the boy is engrossed in (ironically) a popular hospital series. His favorite stuffed animal sits at the foot of his bed. While a diagnosis cannot be based on drawings alone, the first two images certainly raise the question of depression in these children and can furnish an opening in a psychological evaluation or psychotherapy.

**Figure 16:**
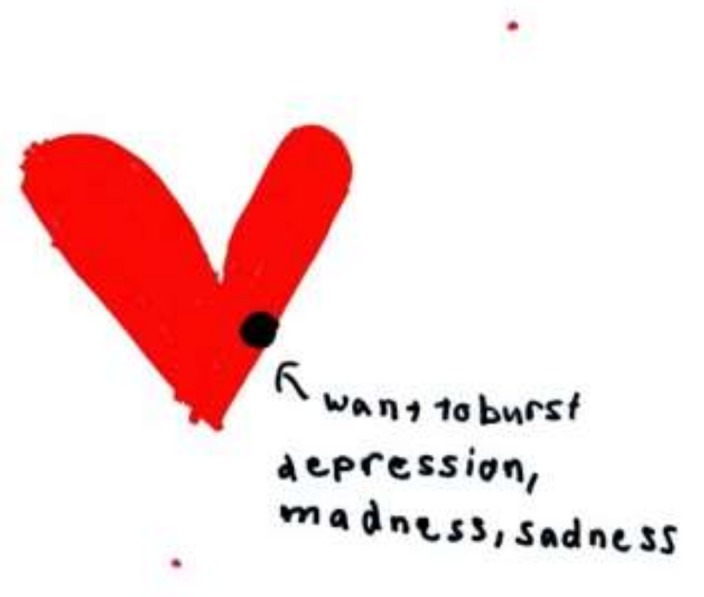
A heart that wants to burst.

Occasionally children will name the symptom explicitly; in Figure 16 [8] (p. 27), a sibling describes the intensity of her feelings regarding the illness of her younger sister and includes depression in the triad of emotions.

**Figure 17:**
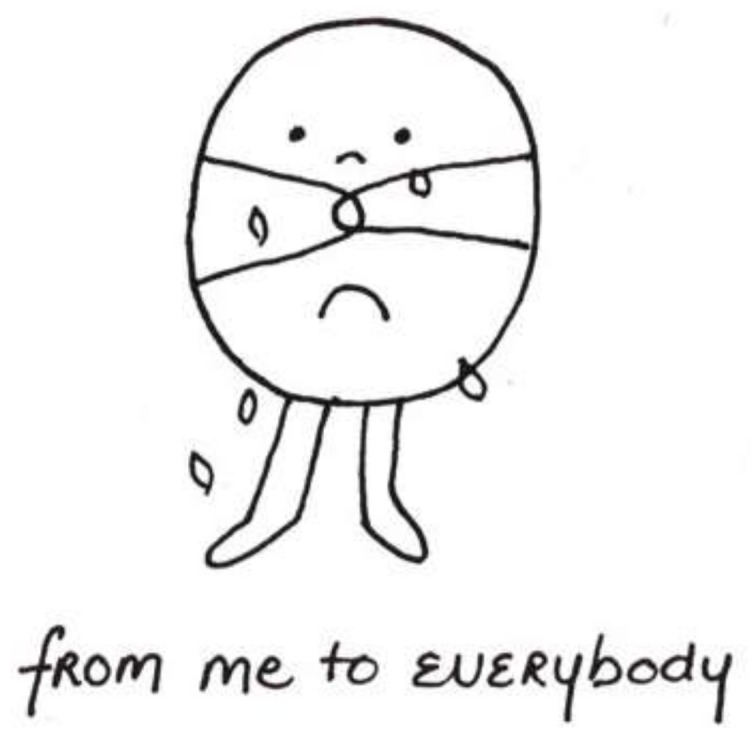
From me to everybody.

Distinguishing the clinical disorder of depression from sadness or anticipatory grief in seriously-ill children can be a poignant challenge. Thus, in Figure 17 [2] (p. 49), a child who had a large tumor in her cheek (thus the bands across the little figure’s face) entitled her implicit self-portrait: “From me to everybody.” The image reflected not depression, but rather her sadness and grief—her separateness from everyone—as she faced death.

**Figure 18:**
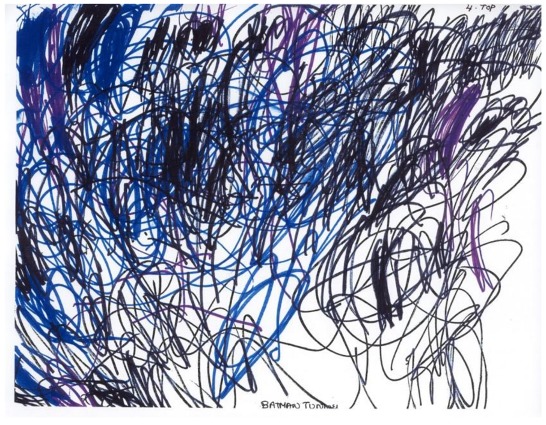
Batman tunnel.

**Figure 19:**
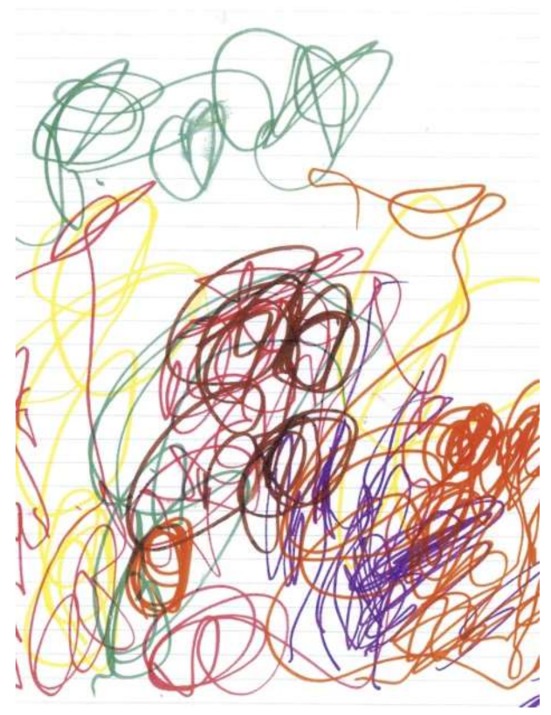
Untitled.

Anxiety can emerge in many contexts, from specific triggers (e.g., procedure related) to a generalized sense of foreboding. It is the latter that can be difficult to identify and address in children. A four-year-old child being cared for in home-hospice would not verbally acknowledge that he had any symptoms of any kind. However, he described a dark and threatening drawing as “a batman tunnel. It’s completely dark, but I wasn’t scared” (Figure 18) [17] (p. 85). This image certainly connotes anxiety or distress—especially in contrast with his earlier bright pictures (Figure 19) [17] (p. 85).

Nightmares are not uncommon manifestations of anxiety. Thus, this interchange:

Psychologist: What are your bad dreams about?

Child: Monsters, a snake biting....

Psychologist: When you have those bad dreams, what do you think you are worried about?

Child: You dying. Everyone dying in the world and leaving me alone....

[2] (p. 122–123)

**Figure 20:**
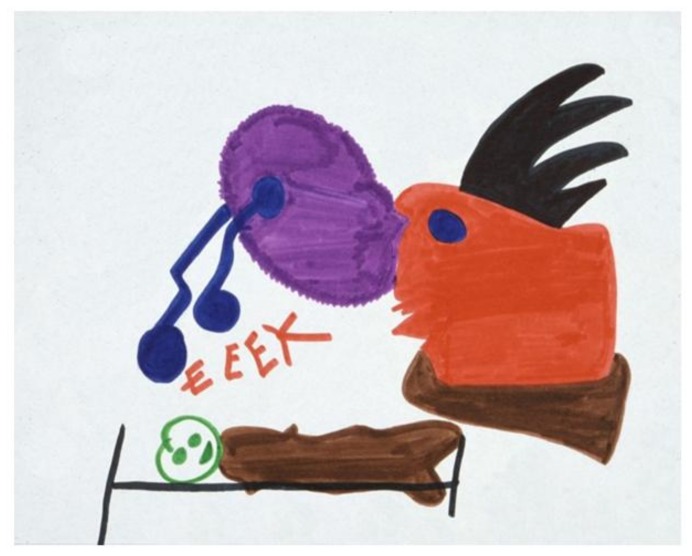
Nightmare.

In a drawing by another child (Figure 20) [2] (p. 123), he portrayed himself in bed, overwhelmed by a grotesque monster who seems to be “breathing terror” toward him. The boldly printed word “EEEK” was the only verbal accompaniment that the child could offer.

## 9. Conclusions

Many children are capable of describing symptoms vividly, whether in words or images, even at times offering an explanation or interpretation as to their significance. These selected narratives underscore the inextricable nature of the physical and psychological in the expression of many symptoms. Their complexity is testimony to the crucial need for an interdisciplinary treatment team and the paramount importance of ongoing communication among the children, families, and professionals who care for them.
